# Dipole Modifiers Regulate Lipid Lateral Heterogeneity in Model Membranes

**Published:** 2017

**Authors:** S. S. Efimova, O. S. Ostroumova

**Affiliations:** Institute of Cytology of the Russian Academy of Sciences, Tikhoretsky av. 4, St. Petersburg 194064 , Russia

**Keywords:** chalcones, flavonoids, lateral heterogeneity, lipid bilayers, lipid domains, dipole modifiers, styrylpyridinium dyes phase separation

## Abstract

In this study we report on experimental observations of giant unilamellar liposomes composed of ternary mixtures of cholesterol (Chol), phospholipids with relatively low T_melt_ (DOPC, POPC, or DPoPC) and high T_melt_ (sphingomyelin (SM), or tetramyristoyl cardiolipin (TMCL)) and their phase behaviors in the presence and absence of dipole modifiers. It was shown that the ratios of liposomes exhibiting noticeable phase separation decrease in the series POPC, DOPC, DPoPC regardless of any high-T_melt_ lipid. Substitution of SM for TMCL led to increased lipid phase segregation. Taking into account the fact that the first and second cases corresponded to a reduction in the thickness of the lipid domains enriched in low- and high-T_melt_ lipids, respectively, our findings indicate that the phase behavior depends on thickness mismatch between the ordered and disordered domains. The dipole modifiers, flavonoids and styrylpyridinium dyes, reduced the phase segregation of membranes composed of SM, Chol, and POPC (or DOPC). The other ternary lipid mixtures tested were not affected by the addition of dipole modifiers. It is suggested that dipole modifiers address the hydrophobic mismatch through fluidization of the ordered and disordered domains. The ability of a modifier to partition into the membrane and fluidize the domains was dictated by the hydrophobicity of modifier molecules, their geometric shape, and the packing density of domain-forming lipids. Phloretin, RH 421, and RH 237 proved the most potent among all the modifiers examined.

## INTRODUCTION

The lipid bilayer may have a domain structure determined by immiscible lipid phases coexisting in different aggregate states. Single-component lipid bilayers exist in the solid state at temperatures below the melting points (T_melt_) of lipids. Depending on the tilt angle of lipid molecules and the packing of hydrocarbon tails, the solid bilayer is comprised of the following phases: the solid phase (crystalline), the gel phase, and the ripple phase, which is typical of saturated phosphocholines [1]. At a point above the transition temperature, the state of bilayer lipids changes into a liquid-like state. Lipid components with varying melting temperatures can show complicated phase behavior in different areas of the membrane in a temperature-dependent manner. This leads to the coexistence of solid (*s_o_*) and liquid states (*l_d_*) attributed to lipids with high and low melting temperatures, respectively. The presence of sterols, in particular cholesterol, promotes phase segregation and induces the liquid-ordered state (*l_o_*). There is evidence that the coefficient of lateral lipid diffusion in the *lo*- phase is 2–3 times lower as compared to the *l_d_*-areas [2]. The existence of lipid lateral segregation has been demonstrated in biological membranes. Although gel domains are not exclusive to model membranes (they are also present in biological membranes [3]), it has been generally assumed that phase segregation in biological membranes is mainly represented by two liquid phases (*l_d_*+ *l_o_*) [4]. Since not only membrane lipids are sensitive to lateral segregation, but also peptides, a concept of lipid-protein nanodomains (rafts) has been proposed and received increasing attention. These rafts are enriched in high-T_melt_ lipids and cholesterol and exist in the *l_o_*o-phase. In recent years there has been growing interest in lipid rafts due to their important role in protein trafficking, signaling, immune response, etc. [5-16]. Importantly, the occurrence of lipid-protein rafts has not yet been agreed upon. These nanodomains are one to hundreds of nanometers in size and are extremely dynamic. In lipid bilayers, the ordered domains can be of large dimension, which allows for visualization by fluorescence microscopy using single unilamellar liposomes [17]. It is possible to observe phase segregation in liposomes loaded with fluorescently labeled lipids. Most dyes are targeted at the liquid-disordered raft fraction, leaving the ordered domains unlabeled.

**Table:** The main characteristics of the lipid molecules

Lipid	Chemical structure	C n:m	T_melt_, °C
DPoPC	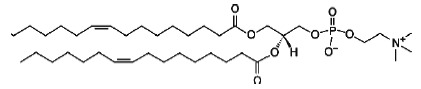	16 : 1	-36
DOPC	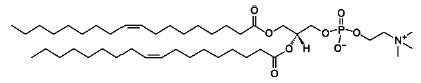	18 : 1	-17
POPC	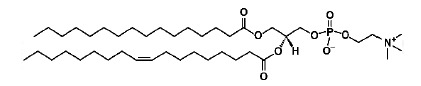	16 : 0–18 : 1	-2
SM	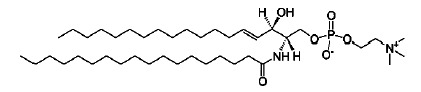	16 : 1–18 : 0	45
TMCL	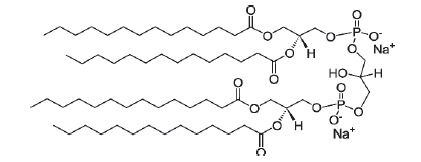	14 : 0	47

Note. C*n *: *m *is the number of carbon atoms (*n*) and double bonds (*m*) in acyl chains; *T_melt_*is the main phase-transition temperature.

Amphiphilic low-molecular-weight compounds, known as dipole modifiers, in particular some flavonoids, can influence the equilibrium between the phases. Ostroumova *et al. *[18] reported that flavonoid compounds such as biochanin A, phloretin, and myricetin are able to negatively affect phase separation scenarios in model membranes composed of binary lipid mixtures (DOPC : SM (80 : 20 mol.%), DOPC : DMPC (50 : 50 mol. %) or DOPC : DPPC (50 : 50 mol. %)). A similar effect was observed for phloretin, its glycoside phlorizin, quercetin, myricetin, and styrylpyridinium dyes of the RH series in a three-component bilayer mixture of POPC, Chol, and SM [19]. Although Efmova *et al. *[19] examined the influence of the above-mentioned dipole modifiers on the domain structure of POPC membranes incorporating sterols and sphingolipids, the roles of these phospholipids, which constitute the disordered liquid phase, remain poorly understood. The objective of this work was to investigate the effect of low-T_melt_ lipid components on the phase separation scenario in liposomes packed with Chol and SM before and after the introduction of flavonoids or RH dyes. With a variety of phospholipids, POPC, DOPC and DPoPC, we were able to sequentially change the disordered lipid phase thickness of a fluid membrane. Lipid mixtures containing TMCL were also studied for their ability to modify the thickness of ordered lipid domains.

## MATERIALS AND METHODS

**Materials**

The following compounds were used in the study: sorbitol, phloretin, phlorizin, quercetin, myricetin, and RH 421 (Sigma, USA); RH 237 (Molecular Probes, USA); 1-palmitoyl-2-oleoyl-sn-glycero-3-phosphocholine 1 (POPC), 1,2-dioleoyl-sn-glycero-3-phosphocholine (DOPC), 1,2-dipalmitoleoyl-*sn*-glycero-3-phosphocholine (DPoPC), 1,1’,2,2’-tetramyristoyl cardiolipin (TMCL), porcine brain sphingomyelin (SM), cholesterol (Chol) and lissamine rhodamine B-1,2-dipalmitoyl-sn-glycero-3-phosphoethanolamine (Rh-DPPE) (Avanti Polar Lipids, USA). The Table provides details for each of the lipids used.

**Fig. 1:**
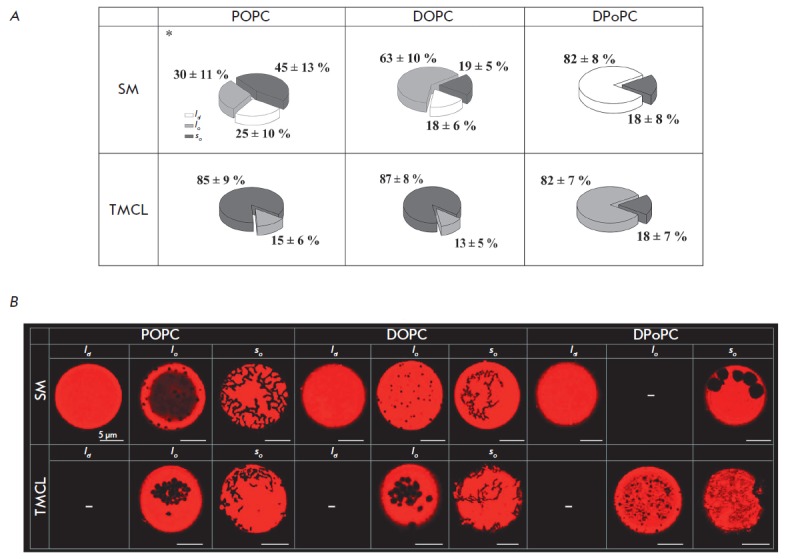
Pie charts demonstrating the possible scenarios of phase separation in liposome membranes composed of sphingomyelin (SM) or tetramyristoyl cardiolipin (TMCL) (40 mol. %), cholesterol (Chol) (20 mol. %), and different phospholipids (POPC, DOPC, or DPoPC) (40 mol. %). **(A) **Microphotographs of liposomes with different lipid compositions and phase behaviors (*l_d_*, *l_o_*, *s_o_*) (B). Here and in *Fig. 3* and *Fig. 4*, dark gray sectors denote the percentage of vesicles with solid ordered domains (*s_o_*); light gray sectors denote the percentage of vesicles with liquid-ordered domains (*l_o_*); white sectors denote the relative number of homogeneously stained liposomes with liquid-disordered state without noticeable phase separation (*l_d_*). * – data from ref. [19]. **(B) **Fluorescence micrographs of giant unilamellar vesicles demonstrating various types of membrane phase separation scenarios (*l_d_*, *l_o_*, *s_o_*).

**Confocal microscopy of giant unilamellar liposomes**

Giant unilamellar liposomes were prepared by the electroformation technique using the Vesicle Prep Pro machine (Nanion, Germany) on glass slides coated with titanium and indium oxides (90% indium oxide : 10% indium oxide, 29 × 68 × 0.9 mm) with a surface specific resistivity of 20–30 Ω/sq. (standard protocol, 3 V, 10 Hz, 1 h, 25°C.) Lateral phase segregation was visualized by adding the Rh-DPPE fluorescent probe into a three-component mixture that consisted of 40 mol. % low-T_melt_ phospholipid (DOPC, POPC or DPoPC), 40 mol. % high- T_melt_ phospholipid (SM or TMCL), and 20 mol. % Chol in chloroform (2 mM). The final Rh-DPPE concentration was 1 mol. %. The liposome suspension was aliquoted for storage. An aliquot without a dipole modifier was used as control. Test samples contained 400 μM flavo noid (phloretin, phlorizin, quercetin, or myricetin) or 10 μM styrylpyridinium dye (RH 421 or RH 237). Images were acquired with APO oil-immersion objective lens 100.0 × /1.4 HCX PL using a Leica TCS SP5 confocal laser scanning microscope (Leica Microsystems, Germany). Liposomes were examined at 25°C. Rh- DPPE emission was excited at 543 nm (a helium-neon laser). There is evidence that in lipid bilayer systems with phase segregation, Rh-DPPE shows partitioning preference mainly for the disordered liquid phase (*ld*) [20], whereas the liquid-ordered phase (*lo*) and solid ordered phase (gel, *so*) remain unlabeled [21]. Ordered domains were identified morphologically: the dye-unlabeled circular domains were considered to be in the *lo*-state, while the dye-unlabeled domains of irregular shape were assigned to the *so*-state. Each sample was characterized by the ratio (*pi*, %) of homogeneous and heterogonous vesicles:

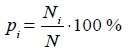



where *i *is liposome phase separation (homogeneous *ld*-vesicles or liposomes that carry the *lo *or *so*-domains); *Ni *is the vesicle number in a sample with a certain phase scenario (from 0 to 50); and *N *is the total liposome number in a sample (50 in each system). The *pi *values were obtained by averaging values from four independent experiments. The data for each lipid system were presented in pie charts, along with standard deviations for liposomes with assigned phase behavior.

## RESULTS AND DISCUSSION

**Fig. 2:**
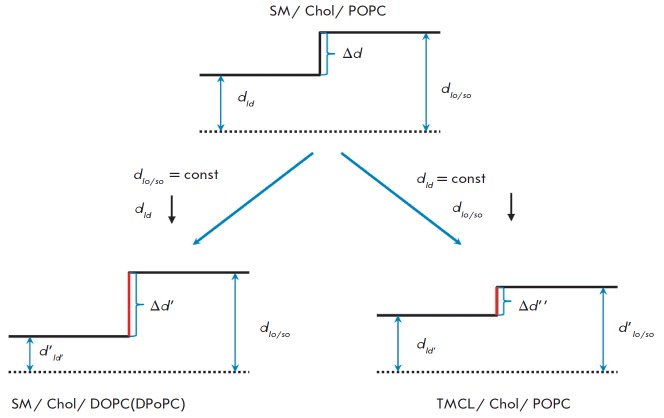
Schematic representation of the correlation between the thickness mismatch (Δ*d*) of the ordered (*d_lo/so_*) and disordered domains (*d_ld_*) and bilayer lipid composition. The dotted line marks the center of the bilayer; the solid line indicates the boundary between the polar and nonpolar regions of the membranes. For color designations, see *Fig*. *1. *

*Fig. 1A* ( *upper panel*) shows findings on possible types of phase behavior in unilamellar membranes comprised of SM (40 mol. %), Chol (20 mol. %), and low- T_melt_ phospholipids (POPC, DOPC or DPoPC; 40 mol. %) (see Table for details on T_melt_). Microphotographs with each type of phase segregation scenario (*l_d_*, *l_o_*, *s_o_*) are presented in *Fig. 1B*. (*upper panel*). Phase behavior of ternary mixtures containing SM/Chol/ POPC had been previously examined [19]. We found that liposomes that incorporate 45 ± 13% SM/Chol/POPC contain solid domains of irregular shape (*s_o_*), whereas 30 ± 11% SM/ Chol/POPC vesicles are enriched in liquid-ordered domains with a circular morphology (*l_o_*). The remaining liposomes are vesicles homogeneously labeled with the fluorescent probe (*l_d_*), exhibiting no phase segregation. *Fig. 1A* (*upper panel*) demonstrates that substitution of POPC for DOPC in the membrane mixture reduces the number of vesicles with *s_o_*-domains (19 ± 4%) and increases the number of liposomes with the *lo*-state (63 ± 10%). When DPoPC was used, 82 ± 8% vesicles were homogeneously dye-labeled without noticeable phase separation, while the remaining vesicles contained solid domains. It is tempting to suggest that visual phase separation decreases in the series POPC, DOPC, DPoPC, and so does the thickness of the disordered phase (*d_Ld_*), which includes different low-T_melt_ phospholipids, whereas the mismatch (Δ*d*) in the hydrophobic bilayer thickness of the coexisting liquid-ordered and liquid-disordered phases increases (*Fig. 2*, *left part*) [22, 23]. As a result, the formation of well-defined boundaries between the ordered and disordered domains, which seemingly favors the exposure of a portion of the hydrophobic region to the aqueous environment, becomes energetically prohibitive, thus decreasing the number of liposomes with visible phase separation. A similar conclusion can be reached based on the results shown in *Fig*. *1 *(*lower panel*), which presents the data on the phase separation of TMCL membranes ((TMCL; 40 mol. %), Chol (20 mol. %) and other low-T_melt_ phospholipids (40 mol. %)). One can notice that TMCL-containing liposomes show phase separation regardless of any low-T_melt_ phospholipids (no homogeneously labeled liposome). The differences between the lipid systems are due to the proportion of vesicles carrying *l_o_*- and *s_o_*-domains. As noted above, DPoPC contributes to the lowest thickness of the *l_d_*-phase among all the phospholipids tested, which corresponds to the highest Δ*d *value, and consequently to the highest energy of ordered domain formation. This explains why DPoPC-containing liposomes showed poor phase separation (82 ± 7% liposome have *l_o_*-domains) versus the POPC-and DOPC-vesicles that form *l_d_*-phases with greater thickness and lower Δ*d *values with phase separation in most liposomes (85 ± 9% and 87 ± 8% in the *l_d_*/*s_o_*ratio, respectively).

An analysis of phase behavior scenarios involving various high-T_melt_ phospholipids also suggests a role for Δ*d *in regulating the lateral heterogeneity of ternary membrane mixtures. *Figure 1B* (*lower panel*) depicts microphotographs of TMCL-containing liposomes with low-T_melt_ phospholipids. *Figure 1A*demonstrates that SM to TMCL substitution in the membrane mixture leads to enhanced phase separation. In the case of POPC- (*left column*) and DOPC-containing bilayers (*middle column*), the proportion of liposomes with *so*- domains increases, whereas DPoPC-bilayers display a statistically significant increase in the numbers of vesicles with *l_o_*-domains (*right column*). This is attributed to the fact that the presence of TMCL in place of SM lowers the thickness of the ordered phase and decreases Δ*d* (*Fig. 2*, *right part*). Taken together, this substitution finally reduces the energy of ordered domain boundary formation. Overall, the findings in *Fig*. *1 *allow one to link the lateral heterogeneity of ternary membranes to the mismatch in the membrane thickness of the liquid-ordered and liquid-disordered phases: the degree of phase separation is inversely proportional to Δ*d *values.

**Fig. 3:**
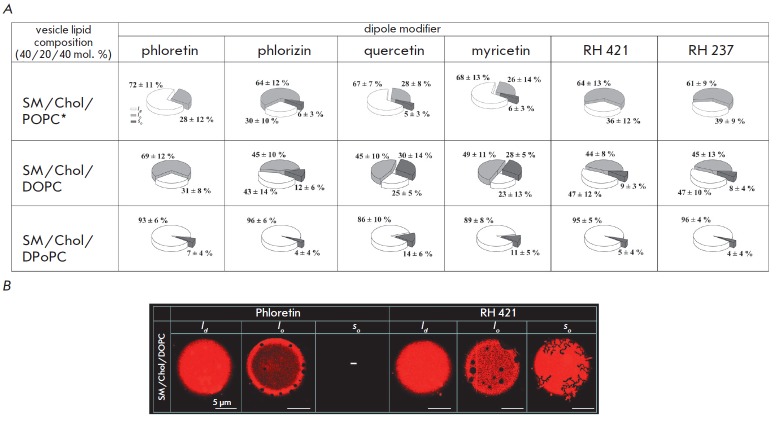
**(A) **Pie charts demonstrating the possible scenarios of phase separation in liposome membranes composed of sphingomyelin (SM) (40 mol. %), cholesterol (Chol) (20 mol. %), and different phospholipids (POPC, DOPC or DPoPC) (40 mol. %) in the presence of dipole modifiers (400 μM phloretin, 400 μM phlorizin, 400 μM quercetin, 400 μM myricetin, 10 μM RH 421, and 10 μM RH 237). For color designations, see *Fig*.1. * – data from ref. [19] **(B) **Fluorescence microphotographs of SM/Chol/DOPC-liposomes demonstrating various types of membrane phase separation scenarios (*l_d_*, *l_o_*, *s_o_*) in the presence of phloretin and RH 421.

Taking into account the fact that dipole modifiers impact not only the dipole potential, but also the packing of lipid components [24-27], we suggest that these agents possess the ability to alter the phase separation scenario. Recently, we have investigated the effects of the dipole modifiers phloretin, phlorizin, quercetin, myricetin, RH 421, and RH 237 on the phase separation behavior in SM/Chol/POPC-vesicles [19]. The data are shown in *Fig*. *3A *(*upper panel*). It is clear that the dipole modifiers decrease membrane phase separation, which manifests itself as reduced liposome numbers with gel domains. However, upon incorporation of phloretin, quercetin, or myricetin, the decline in the number of vesicles with *s_o_*-domains is accompanied by a corresponding 40–45% increase in the number of homogeneously stained liposomes. The presence of phlorizin, RH 421, and RH 237 induced a 30–35% increase in the ratio of vesicles with *l_o_*-domains and a 5–10% increase in the number of homogeneous liposomes. The elevated liposome concentrations versus homogeneously labeled DOPC-liposomes in the presence of phloretin, phlorizin, RH 42,1 and RH 237 (by 10–30%) and elimination of vesicles with *s_o_*-domains in the presence of phloretin could be explained by decreased phase separation following the addition of dipole modifiers as in the case with POPC (*Fig. 3AA*, *middle panel*). *Figure 3B* shows microphotographs of lipid vesicles containing DOPC, Chol, and SM and their phase behaviors (*l_d_*, *l_o_*, *s_o_*) in the presence of phloretin and RH 421. No statistical significance was found regarding the effects of quercetin and myricetin on phase separation in DOPC membranes.

Changes in the phase separation scenario of SM-containing membranes in the presence of dipole modifiers could be caused by elevated Δ*d *values under the influence of the agents tested. The most likely scenario is that the polar heads of lipids take over more space in the membrane in response to burying of the modifiers into the lipid layer and dipole-dipole interactions between them. As shown by differential scanning calorimetry, this relatively increases the mobility of carbohydrate chains and reduces the T_melt_ of lipids [18, 25, 28]. The more “fluid-like” state of the membrane correlates with the decreased bilayer thickness. In this case, the extent of the effect of a modifier will depend on the backbone and overall hydrophobicity, which govern the degree to which the modifier is buried into the bilayer. That is why the hydrophobic phloretin exerts the strongest effect on membrane lateral heterogeneity, whereas its hydrophilic analog, phlorizin, and the highly hydroxylated flavonoids quercetin and myricetin exhibit weaker effects. The length of the styrylpyridinium dyes RH 421 and RH 237 is sufficient to transverse the lipid monolayer, but the increase in the space occupied by a single lipid in the membrane is largely due to electric repulsion among the sulfonate groups located in the polar bilayer region [27].

In addition to the modifier type, the geometric characteristics of lipid molecules that form the phase into which a modifier partitions also play a regulating role. In the case of lipids with a cylindrical geometry, such as DOPC, POPC, and SM [29-31], *l_d_*-domains become sensitive to fluidization as compared to ordered domains, since partitioning of modifiers into the *ld*-domains seems to be impeded in the context of tightly packed lipids. This scenario is schematically illustrated in *Fig*. 2 (*left part*).

As shown by the lower panel in *Fig*. *3A*, DPoPC-containing membranes exhibited no statistically significant differences between phase behavior scenarios before and after the modifiers had been added. Bearing in mind that no phase separation is observed in most DPoPC-vesicles even in the absence of dipole modifiers due to the greatest mismatch in the membrane thickness of the liquid-ordered and liquid-disordered phases, further elevation of Δ*d *does not lead to significant changes in bilayer phase separation.

**Fig. 4:**
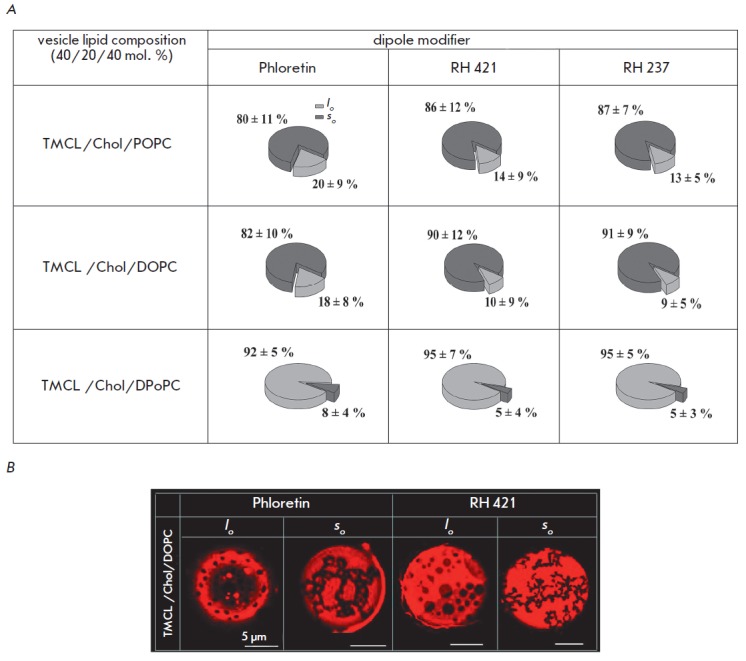
**(A) **Pie charts demonstrating the possible scenarios of phase separation in liposomes membranes composed of tetramyristoyl cardiolipin (TMCL) (40 mol. %), cholesterol (Chol) (20 mol. %), and various phospholipids (POPC, DOPC, or DPoPC) (40 mol. %) in the presence of dipole modifiers (400 μM phloretin, 10 μM RH 421, and 10 μM RH 237). For color designations, see *Fig*.1. **(B) **Fluorescence micrographs of TMCL/Chol/DOPC-vesicles demonstrating *l_d_/l_o_*- or *l_d_/s_o_*-phase separation in the presence of phloretin and RH 421.

In contrast to SM, TMCL has an inverted cone shape that triggers inverted spontaneous curvatures of the monolayers formed by it [32]. It is highly likely that this favors partitioning of dipole modifiers having a cone shape into the ordered TMCL-enriched phase. Simultaneous fluidization of disordered *l_d_*-domains and ordered domains will not dramatically alter the thickness mismatch between the phases, thus preventing changes in phase behavior scenarios. *Figure 4A* shows that regardless of the type of low-T_melt_ lipid within the model membranes, the presence of a dipole modifier neither significantly increases the relative number of TMCL-containing liposomes with *l_o_-*domains nor induces the emergence of liposomes with noticeable phase separation. *Fig. 4B* shows mi crophotographs of lipid vesicles incorporating DOPC, Chol, and TMCL and liposomes modified with phloretin or RH 421.

In conclusion, our findings suggest a key role for the mismatch thickness between the ordered and disordered phases in modulating phase behavior scenarios in ternary model membranes. It is believed that our work will open up new a venues for research into the use of dipole modifiers for the regulation of lipid lateral heterogeneity in bilayers.

## Abbreviations

Chol: cholesterol
DOPC: 1,2-dioleoyl-sn-glycero-3-phosphocholine
DPoPC: 1,2-dipalmitoleoyl-sn-glycero-3-phosphocholine
POPC: 1-palmitoyl-2-oleoyl-sn-glycero-3-phosphocholine
SM: porcine brain sphingomyelin
TMCL: 1,1’,2,2’-tetramyristoylcardiolipin
